# Breastfeeding Practices among Mothers in Southwest Nigeria

**DOI:** 10.4314/ejhs.v30i5.8

**Published:** 2020-09

**Authors:** Adebayo Akadri, Oluwaseyi Odelola

**Affiliations:** 1 Department of Obstetrics and Gynaecology, Babcock University, Ilishan-Remo, Ogun State, Nigeria; 2 Department of Obstetrics and Gynecology, Olabisi Onabanjo University Teaching Hospital, Sagamu, Ogun State, Nigeria

**Keywords:** Breast milk, Breastfeeding, exclusive breastfeeding, Nigeria, Practices

## Abstract

**Background:**

This study was conducted to determine the breastfeeding practices among parous antenatal attendees in two teaching hospitals in Southwest Nigeria.

**Methods:**

A cross-sectional study was carried out on 340 parous antenatal clinic attendees in two teaching hospitals in Ogun State, Nigeria. A structured questionnaire was used to obtain data on breastfeeding practices. Data were analyzed using Statistical Package for Social Sciences (SPSS) windows version 21.0 (IBM Corp., Armonk, NY, USA).

**Results:**

Out of a total of 340 women, 319 (93.8%) breastfed their last babies. The median duration of breastfeeding was 15 months. One hundred and thirty two women (38.8%) initiated breastfeeding within 30 minutes of delivery, and 200 women (58.8%) exclusively breastfed their babies for six months. The majority of the women, 299(87.9%k) did not report any problem associated with breastfeeding. Logistic regression analysis showed that women who had no breastfeeding problems were more likely to exclusively breastfeed their infants for six months compared to those with breastfeeding problems (AOR 3.4; CI 1.6–7.1; P=0.001). Similarly, women who got breastfeeding information from mass media were more likely to practice exclusive breastfeeding for six months compared to those that got breastfeeding information from other sources (AOR42.2; CI 3.1–568.5; P=0.005).

**Conclusion:**

Exclusive breastfeeding is suboptimal in Ogun State, Nigeria. Provision of breastfeeding information via mass media will assist in improving breastfeeding practice. Educating mothers on the techniques that help to prevent breastfeeding complications will also be beneficial.

## Introduction

Adequate nutrition is essential for optimal growth and development of infants. Breast milk is natural and contains all the necessary nutrients required by infants in the first six months of life (1). In addition to being an important source of energy and nutrients, breast milk also has immunological properties and thus lowers the chance of infants becoming ill or dying from infections such as gastroenteritis, pneumonia and meningitis (2,3). It has been estimated that optimal breastfeeding could reduce under-five mortality worldwide by as much as 823,000 each year (4). The beneficial effects of breastfeeding extends beyond infancy. Evidence suggests that breastfeeding protects against childhood obesity, and adolescents who were breastfed as babies were likely to be more intelligent than those who were not breastfed (3). Research has also shown that breastfeeding is possibly protective against chronic noncommunicable diseases such diabetes and hypertension (5).

The World Health Organization (WHO) and the United Nations Children's Fund (UNICEF) have identified breastfeeding as the single most effective and affordable feeding practice necessary for development of healthy infants (6). These organizations have thus recommended initiation of breastfeeding within one hour of birth, exclusive breast feeding for the first six months of life and continued breastfeeding up to two years of age or beyond (3).

Exclusive breastfeeding is when only breast milk is given to the child, except for medicines, vitamins or mineral supplements (7). There appears to be universal awareness about exclusive breastfeeding among women in many countries; however, breastfeeding practices are often not aligned with recommendations (8,9). Globally, only 43% of newborns have breastfeeding initiated within one hour of birth, and 40% of infants aged six months or less are exclusively breastfed (3). In sub-Saharan Africa, studies have shown that the prevalence of early initiation of breastfeeding ranges from 37.8% to 69.3% while the prevalence of exclusive breastfeeding ranges from 23.7% to 56.5% (10). A recent national survey suggested that only 29% of infants less than six months were exclusively breastfed in Nigeria (11). There is no data on the average duration of exclusive breastfeeding in south west Nigeria; however, over 70% of women in southwest Nigeria breastfeed their infants for up to a year (9).

Breastfeeding practices may be influenced by several factors such as psychosocial, cultural, economic and health factors (9). In a study done in Ghana, the educational status, age and ethnicity of women were found to be strong predictors of maternal practice of exclusive breastfeeding (12). Access to breastfeeding counseling during pregnancy, vaginal mode of delivery and support by husband are also factors that positively influence the initiation and maintenance of breastfeeding (2,13,14). The occupation and work schedule of nursing mothers often times act as barriers to optimal breastfeeding (15,16). Women who resume work before their babies are six months old will likely find it difficult to adhere to the recommendation of exclusive breastfeeding (15). The provision of nursing areas in the work environment may assist nursing mothers in improving breastfeeding practices. Support to breastfeeding mothers in the form of job flexibility and rearrangement of work schedule may also enable them to breastfeed successfully (15,16).

Optimal breastfeeding practices are linked to achieving global goals. The second sustainable development goal (SDG) focuses on improving nutrition and ending hunger while the third SDG aims at ensuring healthy lives and promoting the well-being of individuals of all ages (6). It is clear that the promotion of breastfeeding in all countries will be an important strategy in achieving these goals among infants and young children (6).

Breastfeeding practices are often culture dependent; hence, a proper understanding of the factors that influence them in any particular cultural setting is important in designing successful interventions. This study is aimed at determining the breastfeeding practices among parous antenatal attendees in two tertiary hospitals in Southwest Nigeria.

## Materials and Methods

**Study setting and design**: This cross-sectional study was carried out at the antenatal clinics of Babcock University Teaching Hospital (BUTH), and Olabisi Onabanjo University Teaching Hospital (OOUTH). Both hospitals are in Ogun State, Nigeria. The target population included all pregnant women attending antenatal clinic at the hospitals who had practiced breastfeeding in previous pregnancy.

**Sample size and sampling technique**: The minimum sample size for the study was determined using the Leslie-Kish formula (17): (n = Z^2^pq/d^2^), where n is the desired sample size; Z is the standard normal deviate set at 1.96 (for 95% confidence interval); p is the prevalence of exclusive breastfeeding in the target population; q =1-p and d is the degree of accuracy desired, set at 0.05. An extrapolation from the Nigerian demographic and health survey (11) suggests a prevalence of breast feeding of 27.8%; hence, the calculated sample size was 308. By considering a 10% attrition rate, the overall minimum sample size was 339. Three hundred and forty eligible pregnant women were recruited for the study. These women were recruited consecutively from each study center until the estimated sample size was obtained.

**Data collection**: A structured questionnaire was used to obtain information on the sociodemographic characteristics of each study participant such as the age, parity, religion and level of education. The women were also asked about the place where they had antenatal care (ANC) in the last pregnancy and where they had their last delivery (hospital, mission centre, traditional birth attendants (TBA), home). Information on the initiation, duration and pattern of breastfeeding following the last delivery was also recorded. Breastfeeding practices were assessed by asking the women the time interval between delivery and initiation of breastfeeding, the duration of exclusive breastfeeding, and the total duration of breastfeeding. The problems encountered by the women during breastfeeding were assessed by asking questions regarding the presence or absence of cracked nipples and breast engorgement. Women who reported poor milk production during breastfeeding were said to have inadequate lactation. The sources of information regarding breastfeeding (health workers, mass media, family) were also recorded on the questionnaire.

**Statistical analysis**: Data were analyzed using Statistical Package for Social Sciences (SPSS) windows version 21.0 (IBM Corp., Armonk, NY, USA). Continuous variables were summarized using descriptive statistics. The age of study respondents was summarized using mean and standard deviation at 95% confidence interval. The parity, duration of exclusive breastfeeding and total duration of breastfeeding of the study respondents were summarized using median and interquartile range. Categorical variables were summarized using frequencies and percentages. Pearson's Chi-square test was used to establish the association between some respondents' factors and practice of exclusive breastfeeding. All the significantly associated variables were entered into a multivariate logistic regression model to determine probable determinants of the practice of exclusive breastfeeding. The reference variables were ‘TBA’ for site of antenatal care in last pregnancy; ‘home’ for place of last delivery; ‘family’ for source of breastfeeding information; and ‘yes’ for lactational problems. The model explained 16.0% (Nagelkerke R2) of the variance in exclusive breastfeeding and correctly classified 65.9% of cases. Statistical significance was set at *P* value less than 0.05.

**Ethical considerations**: Ethical approval for the study was obtained from the Babcock University Health Research Ethics Committee (BUHREC). The research was conducted in accordance with the World Medical Association Declaration of Helsinki. The study participants were given adequate information on the study, and those who agreed to participate in the study signed a written consent form. The study participants were assured of the confidentiality of data obtained from them. The data set was anonymised to ensure privacy.

## Results

Three hundred and forty women participated in the study. The mean age (SD) of the study participants was 32.9(4.8) years. The majority, 220(64.7%) were within the 30–39 age range, and 241(70.9%) had a parity ≤ 2. The median parity was 2 with interquartile range of 1–3. Two hundred and seventy two study participants (80%) were Christians, and the majority, 172 (50.6%) had tertiary level education (Table 1).

**Table 1:** Socio-demographic characteristics (N=340)

Sociodemographic characteristics	Frequency (%)
**Age**	
20–29	94 (27.6)
30–39	220 (64.7)
≥40	26 (7.6)
**Parity**	
≤2	241 (70.9)
>2	99 (29.1)
**Religion**	
Christianity	272 (80)
Islam	68 (20)
**Level of Education**	
Primary	16 (4.7)
Secondary	152 (44.7)
Tertiary	172 (50.6)

A total of 319 women (93.8%) breastfed their last babies. The median duration of breastfeeding of the study participants was 15 months with interquartile range of 12 – 18 months. One hundred and thirty two women (38.8%) initiated breastfeeding within one hour of delivery, and 200 women (58.8%) exclusively breastfed their babies for six months. The median duration of exclusive breastfeeding was 6 months with interquartile range of 3 – 6 months. The majority of the women, 246 (72.4%), continued breastfeeding beyond one year. Inadequate lactation was the most common problem associated with breastfeeding, affecting 22(6.5%) of the women; the majority of the women did not report any problem associated with breastfeeding 299(87.9%).Two hundred and sixty eight study participants (78.8%) received information about breastfeeding from health workers, 56 (16.5%) received information from family and friends while 16(4.7%) received breastfeeding information from mass media (Table 2).

**Table 2:** Breast feeding practices among study participants

Breast feeding practices	Frequency (n =340)	%
**Breastfed their last baby**		
Yes	319	93.8
No	21	6.2
**Initiation of breastfeeding** **(Hours)**		
< 1	132	38.8
1 – 2	69	20.3
2 – 24	41	12.0
24– 48	43	12.7
>48	43	12.7
Can't remember	12	3.5
**Duration of exclusive** **breastfeeding (Months)**		
<1	72	21.2
2	12	3.5
3	13	3.8
4	17	5.0
5	26	7.7
6	200	58.8
**Breastfeeding beyond 1 year**		
Yes	246	72.4
No	94	27.6
**Problems associated with** **breastfeeding**		
None	299	87.9
Inadequate lactation	22	6.5
Cracked nipple	17	5.0
Breast engorgement	2	0.6
**Sources of breastfeeding** **information**		
Health workers	268	78.8
Family and friends	56	16.5
Mass media	16	4.7

The association between selected predictor variables and the practice of exclusive breastfeeding is depicted in Table 3. The type of health facilities where antenatal clinic and delivery were held in last pregnancy were shown to have statistically significant association with the practice of exclusive breastfeeding for six months (*P*= 0.001; 0.004 respectively). Similarly, other factors such as the source of information on breastfeeding and the absence of problems with lactation also had statistically significant association with the practice of exclusive breastfeeding for six months (*P*=0.003; 0.004 respectively). The age, parity and education of the study participants had no association with the practice of exclusive breastfeeding.

**Table 3:** Association between predictor variables and the practice of exclusive breastfeeding for 6 months

Predictor variables	Exclusive breastfeeding		*P* value
	Yes (n=200)	No(n=140)	
**Age (Years)**			
20–29	55(27.5)	39(27.9)	0.990
30–39	130(65.0)	90(64.3)	
≥40	15(7.5)	11(7.8)	
**Parity**			
≤2	148(74.0)	93(66.4)	0.146
>2	52(26.0)	47(33.6)	
**Education**			
Primary	5(2.5)	11(7.9)	0.061
Secondary	89(44.5)	63(45.0)	
Tertiary	106(53.0)	66(47.1)	
**Facility where ANC was held last pregnancy**			
Hospital	198(99.0)	126(90.0)	0.001*
Mission centre	1(0.5)	4(2.9)	
TBA	1(0.5)	10(7.1)	
**Facility where last delivery was conducted**			
Hospital	184(92.0)	115(82.2)	0.004*
Mission centre	6(3.0)	6(4.3)	
TBA	1(0.5)	10(7.1)	
Home	9(4.5)	9(6.4)	
**Source of information on BF**			
Health worker	162(81.0)	106(75.7)	0.003*
Family &friend	24(12.0)	32(22.9)	
Mass media	14(7.0)	2(1.4)	
**Lactation problems**			
None	185(92.5)	114(81.4)	0.004*
Yes	15(7.5)	26(18.6)	

The significantly associated variables were entered into a multivariate logistic regression model (Table 4), and analysis showed that women who got breastfeeding information from mass media were more likely to practice exclusive breastfeeding for six months when compared to those that got breastfeeding information from other sources (AOR42.2; CI 3.1–568.5; *P*=0.005). Similarly, women who had no breastfeeding problems were more likely to exclusively breastfeed their infants for six months compared to those with breastfeeding problems (AOR 3.4; CI 1.6–7.1; *P*=0.001).

**Table 4:** Logistic regression analysis of predictor variables of exclusive breastfeeding for 6 months

Predictor variables	Exclusive breastfeeding		AOR	CI	*P* value
	YES	NO			
	(n=200)	(n=140)			
**ANC site in last pregnancy**					
Hospital	198(99.0)	126(90.0)	14.6	0.9–251.7	0.064
Mission centre	1(0.5)	4(2.9)	4.7	0.2–129.2	0.363
TBA	1(0.5)	10(7.1)	1.0		
**Place of last delivery**					
Hospital	184(92.2)	115(82.2)	0.8	0.3–2.4	0.657
Mission centre	6(3.0)	6(4.3)	0.4	0.1–2.1	0.267
TBA	1(0.5)	10(7.1)	0.2	0.01–4.9	0.343
Home	9(4.5)	9(6.4)	1.0		
**Source of information on BF**					
Health workers	162(81.0)	106(75.7)	1.5	0.8–3.0	0.201
Mass media	14(7.0)	2(1.4)	42.2	3.1–568.5	0.005*
Family	24(12.0)	32(22.9)	1.0		
**Lactation problem**					
None	185(92.5)	114(81.4)	3.4	1.6–7.1	0.001*
Yes	15(7.5)	26(18.6)	1.0		

* P≤ 0.05

## Discussion

This study assessed the breastfeeding practices among parous antenatal attendees in two tertiary hospitals in Southwest Nigeria. Findings indicate that more than 90% of the respondents engaged in breastfeeding and the majority continued breastfeeding for between 12 to 18 months. However, fewer than 40% of the women initiated breastfeeding within one hour of delivery while fewer than 60% exclusively breastfed their babies for six months. This could indicate that although breastfeeding is widespread among women in southwest Nigeria, the overall practice does not align adequately with the WHO recommendations.

Optimal breastfeeding is a simple, cost effective way of improving the nutrition and well being of infants and young children. This is particularly important in many low and middle income countries where there appears to be a higher burden of malnutrition and micronutrient deficiencies (8). Breastfeeding is a culturally accepted practice in many ethnic groups in Nigeria (9,18,19). Studies also indicate a high level of awareness and knowledge about breastfeeding among women in south west Nigeria (9,15,20,21). This may have been responsible for the high proportion of respondents who engaged in some form breastfeeding. Similar findings were reported by other researchers (22,23).

The median duration of breastfeeding was 15 months and this was similar to 15.1 months and 14.6 months reported in Edo state, South-South Nigeria and Abuja, federal capital territory, Nigeria respectively (24,25). The variations in breastfeeding duration in different regions of Nigeria may reflect some differences in socio-cultural practices (23). Other factors such as occupation and socioeconomic status of women may impact on the duration of breastfeeding (22). Women with highly skilled occupations are likely to have demanding work schedules, thus unable to breastfeed for long periods (23,26).

Only 38.8% of respondents in this study initiated breastfeeding within the first hour of birth. This is similar to rate of 39.9% reported from a study done in Ghana (27), but lower than 59.2% and 52.6% documented by a studies in Lagos and Ethiopia (2,18). Some of the reasons commonly cited for late initiation of breastfeeding include: poor milk production, caesarean delivery, baby's refusal to suck and aversion for colostrum (which is believed to be unclean by some cultures) (23,28). Early initiation of breastfeeding is very important because the initial milk produced (colostrum) is rich in immunoglobulins which protects the newborn until his/her immune system starts to function (2). Early initiation of breastfeeding is also important in nurturing maternal-infant bonding (29). Appropriate education of women and the provision of supportive environment in places of delivery will help address most of the issues relating to late initiation of breastfeeding.

Breastfeeding was continued beyond one year in more than 70% of the women. This is similar to findings from a previous study (9) done in south western Nigeria which reported that 73% of currently breastfeeding mothers intended to breastfeed for up to a year. It is however not uncommon for women to have the intention of breastfeeding adequately but not able to do so because of difficulties experienced during the process (9,30). In this study, inadequate lactation was the most frequently identified lactation problem, and this was reported in 6.5% of the respondents. This is similar to findings from another study in south west Nigeria (9). The economic challenges presently being experienced in Nigeria may also contribute to reduction in duration of breastfeeding since mothers may be forced to return to full time work early. Workplace fatigue and the lack of conducive environment for breastfeeding in many places of work may also contribute to early weaning (31). Provision of work-time breaks and on-site rooms for breastfeeding are important strategies that may enable more women to breastfeed for longer periods (31).

There are numerous benefits of exclusive breastfeeding; evidence suggests however that the practice is suboptimal even in low resource countries like Nigeria (9). The median duration of exclusive breastfeeding was six months. The proportion of women who engaged in exclusive breastfeeding for the recommended six months was however 58.8%. A previous study in Sagamu, south west Nigeria had reported a similar prevalence of exclusive breastfeeding of 56.1% among mothers of children less than 2 years old (22). Other researchers in south west Nigeria reported exclusive breastfeeding rates ranging from 11% to 86.2% (9,15,32). The differences in study settings (urban or rural), the employment status and work demands of the women may be partly responsible for the wide variations in the rates of exclusive breastfeeding (2,9,20).

Several demographic, cultural, psychosocial and maternal health factors have also been reported to have association with the initiation of breastfeeding, duration of breastfeeding and the practice of exclusive breastfeeding (2,9,12,33). In this study maternal age had no association with the practice of exclusive breastfeeding. Similar finding was reported in a previous study done in Sagamu (22).There are conflicting reports on the influence of maternal educational status on breastfeeding practice (12,23,33–35). In this study maternal educational status had no association with the practice of exclusive breastfeeding; similar finding was reported by researchers in Sokoto, north-west Nigeria (28). In Enugu, south-east Nigeria, mothers that attained tertiary level of educational were found to be more likely to breastfeed exclusively when compared with those with lower educational attainment (33). Similar finding was reported by Ogbo et al (34). It is assumed that women with high level of education probably had better knowledge about breastfeeding and this led to better breastfeeding practice (35). However, some other researchers have reported an inverse relationship between maternal educational attainment and breastfeeding practice (12,23). These researchers argued that women with high educational attainment usually engage in formal employments with busy schedules which negatively impacts on their ability to breastfeed (12,23).

The majority of the study respondents had their antenatal care and deliveries in health facilities and such women were significantly more likely to practice of exclusive breastfeeding for six months. Similar findings were reported by Agbo et al and this may indicate that appropriate information about breast feeding was being passed to women by health workers during antenatal visits (35). More than three quarters of the study participants received breast feeding information from health workers in health facilities where they had their antenatal care and deliveries. Similar findings were reported by other researchers (26,33). The sources of breastfeeding information had statistically significant association with the practice of exclusive breastfeeding for six months; similar findings were reported by researchers in Enugu, south-east Nigeria (33). Although fewer than 5% of the study respondents received breastfeeding information through mass media, these women were significantly more likely to practice exclusive breastfeeding for six months. This may indicate that the media may also be a good source of breastfeeding information (33). Reports from some researchers also suggest that receiving breast feeding information via the mass media leads to early initiation of breast feeding (31).

About 12% of women in this study reported breastfeeding problems such as breast engorgements, cracked nipples and inadequate lactation. These problems may be the consequence of poor breastfeeding positioning and improper latching (36).

In this study, women without any lactation problems were significantly more likely to practice exclusive breast feeding. The integration of routine lactation consultation into early postpartum visits, and adequate counseling on proper techniques of breastfeeding will help to prevent these lactation problems and improve the practice of breastfeeding (37).

This study has some limitations. One is the potential for recall bias because mothers had to remember the details of breastfeeding practices following their last delivery, and the capacity to give correct information may depend on the time interval since last delivery. Also, the fact that the study was conducted in tertiary hospitals meant that results may not be a full reflection of the practice in southwest Nigeria as many women have their deliveries at home, with traditional birth attendants, and in primary and secondary health centers.

In conclusion, exclusive breastfeeding is suboptimal in Ogun State, south west Nigeria. Provision of breastfeeding information via mass media and teaching mothers the techniques that help to prevent breastfeeding complications will assist in improving breastfeeding practice. The prioritization of these interventions to encourage breastfeeding will be useful in the global quest to achieve the sustainable development goals.
